# Mpox outbreak in Africa: Dermatologic implications of rapid vaccine administration

**DOI:** 10.1016/j.jdin.2024.08.009

**Published:** 2024-09-07

**Authors:** Anna Ecanow, June Y. Moon, Jia C. Gao, Alina Zufall, Shoshana Marmon

**Affiliations:** a New York Medical College, Valhalla, New York; b Dermatology Department, NYC Health + Hospitals/Metropolitan Hospital Center, New York, New York; c Dermatology Department, NYC Health + Hospitals/South Brooklyn Health, Brooklyn, New York

**Keywords:** Africa, intradermal, keloids, monkeypox, mpox, *Orthopoxvirus*, smallpox, subcutaneous, vaccination

*To the Editor:* On August 14th, 2024, the World Health Organization (WHO) designated the ongoing mpox (formerly monkeypox) outbreak a global public health emergency.^1^ The mpox strain currently surging in Central Africa appears to be more lethal and may be more easily transmitted through routine close contact, particularly impacting children.^1^ A similar WHO declaration in 2022 involved a less virulent West African strain that spread globally, mainly affecting men who have sex with men and manifesting as painful pustular lesions in the genital region.^1^^,^^2^ Behavioral change and vaccination among high-risk groups was generally effective in stemming the outbreak. However, as global cases declined, rising numbers in Africa were largely overlooked, leaving the continent without sufficient resources to control the spread.

Modified Vaccinia Ankara, a replication-deficient smallpox vaccine administered subcutaneously in a 2-dose series, is used for mpox prevention and postexposure prophylaxis.^2^ The Africa Centers for Disease Control and Prevention (CDC) estimates that more than 10 million doses are urgently needed with negotiations underway for the rapid delivery of 200,000 doses.^1^ To stretch the limited supply during the 2022 outbreak, the U.S. Food and Drug Administration (FDA) issued an Emergency Use Authorization (EUA) to allow for intradermal (ID) inoculation which uses a smaller dose (0.1 mL) of the vaccine compared with subcutaneous (SC) dosing (0.5 mL).^2^ Efficacy of this approach is supported by a phase 2 study demonstrating that ID administration is immunologically noninferior to SC delivery.^2^ However, ID dosing is associated with higher rates of localized skin reactions, including erythema, itching, induration, swelling, and persistent nodules.^2^ Data regarding cutaneous adverse effects of ID inoculation with Modified Vaccinia Ankara in patients with skin of color is limited; the majority of study participants (358) were non-Hispanic White, with 10% Black, and 4% Asian.^2^

Given the heightened risk of adverse skin reactions, the CDC recommends against ID vaccination for individuals with a history of keloids, highlighting the need for sufficient vaccine supplies to support SC administration.^3^ Keloids are 15 times more common in dark skin types, with an incidence of approximately 16% reported in the Democratic Republic of the Congo, the epicenter of the current outbreak, rendering this guidance particularly crucial in Africa.^4^

ACAM2000, a second-generation smallpox vaccine in surplus in the United States, was discussed as an alternative to address the vaccine shortfall during the 2022 outbreak.^2^ However, this vaccine was rejected due to its higher risk of adverse effects.^2^ It is also particularly hazardous for individuals with keloids because it is administered with a bifurcated needle using a multiple puncture technique that elicits localized blistering and scarring.^2^

Another consideration is the interim adoption of a single 0.5-mL SC dose in lieu of the 2-dose regimen. In a recent real-world study, this approach was demonstrated to significantly lower the risk of mpox infection in a high-risk cohort.^5^ Nevertheless, ID inoculation remains the most efficient way to immediately expand limited capacity. Dermatologists should underscore the increased potential for cutaneous adverse effects with this approach, which may be overlooked during an urgent vaccine rollout. Moreover, vigilant surveillance and reporting are essential to accurately assess the incidence and severity of keloids associated with ID vaccination in susceptible populations.

## Conflicts of interest

None disclosed.
